# Case Report: Children with Severe Nutritional Rickets in the Naga Region in Northwest Myanmar, on the border with India

**DOI:** 10.4269/ajtmh.20-1431

**Published:** 2021-06-07

**Authors:** Hein Aung, Kyaw Soe, Frank F. Smithuis, Thomas Lamb, Moe Wint Aung, Frank M. Smithuis

**Affiliations:** 1 Medical Action Myanmar, Yangon, Myanmar;; 2 Myanmar Oxford Clinical Research Unit, Yangon, Myanmar;; 3 Department of Radiology, Amsterdam UMC Imaging Center, Amsterdam, The Netherlands;; 4 Department of Endocrinology, Yangon General Hospital, Yangon, Myanmar

## Abstract

Rickets is an often-neglected, painful, and disabling childhood condition of impaired bone mineralization. In this case series we describe a cluster of 29 children with severe, painful bone deformities who live in the very remote region of Nagaland in northwest Myanmar. Children were found to have low 25-hydroxyvitamin D, elevated parathyroid hormone, and elevated alkaline phosphatase levels, consistent with nutritional rickets secondary to vitamin D deficiency, calcium deficiency, or a combination of the two. After treatment with vitamin D_3_ and calcium carbonate, significant improvement was seen in symptoms, biochemistry, and radiography. This is the first report of nutritional rickets in Myanmar in more than 120 years. Vitamin D and calcium supplementation, and food fortification for pregnant women and young children may be required to prevent this potentially devastating disease.

## INTRODUCTION

Rickets is a disabling childhood condition that results from impaired bone mineralization at the growth plates. It is characterized by skeletal deformity, stunted growth, bone pain, and muscle weakness.^1^ Untreated rickets can result in failure to thrive, developmental delay, lifelong skeletal deformity, obstructed labor, and osteomalacia.^1,2^ Vitamin D deficiency and/or calcium deficiency are the most common causes of rickets.^3^ Vitamin D deficiency is often the result of insufficient sunlight exposure in combination with inadequate vitamin D intake. Although insufficient sun exposure is not expected in (sub-) tropical countries, it can be caused by particular sociocultural or religious behaviors.^4^ In Yangon, the commercial capital of Myanmar, vitamin D insufficiency (< 50 nmol/L) was reported in 49 of 60 (82%) health-care workers (M. W. Aung, personal communication). However, this was attributed to a habit of sun avoidance for aesthetic purposes as a pale skin is considered desirable. Children in rural areas are generally expected to have sufficient sun exposure. Inadequate vitamin D intake can be caused by maternal vitamin D deficiency, exclusive breastfeeding, and a vitamin D–poor diet (vegetarian and/or lack of vitamin D–enriched food).^5^ Calcium deficiency is caused predominantly by poor access to dietary products after weaning. We report a cluster of children with severe rickets in Nagaland, northwest Myanmar.

## CASE REPORT

**Figure 1.**
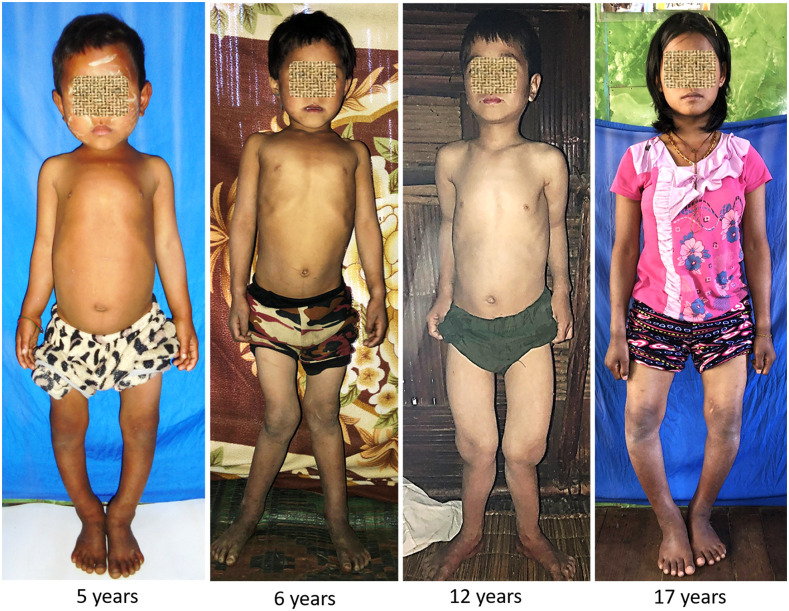
Photographs of children demonstrating typical features of genu valgus, genu varum, wrist swelling, and rachitic rosary before treatment.

Medical Action Myanmar (MAM) is a medical aid organization that provides basic medical care to 275 villages in Nagaland, a remote mountainous region in the far northwest of Myanmar, on the border with India. In January 2019, MAM mobile medical teams identified 29 children in nine villages in Nanyun township (lat. 27°N) with painful joints, walking difficulties, and knee and wrist joint deformities clinically consistent with rickets (Figure 1).

**Figure 2.**
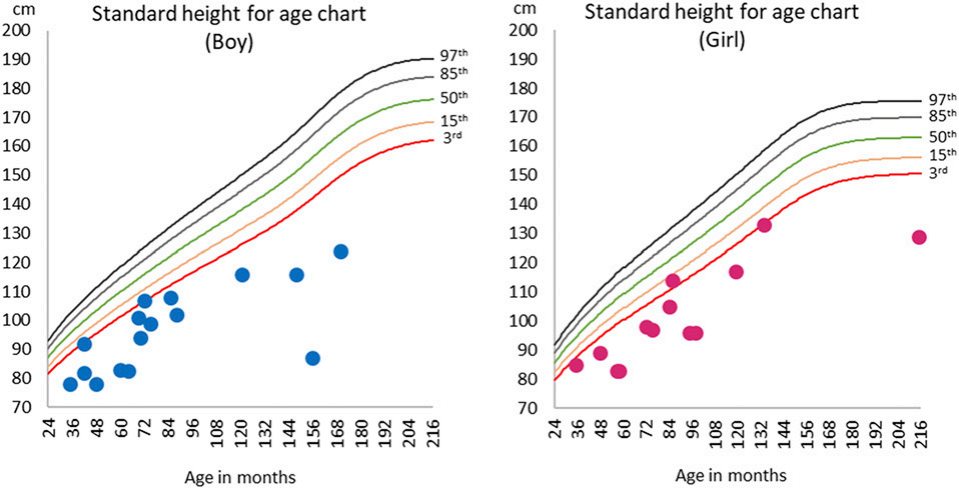
Standard child height-for-age charts, differentiated for girls and boys. The red and blue dots represent observed patient data at baseline.

**Figure 3.**
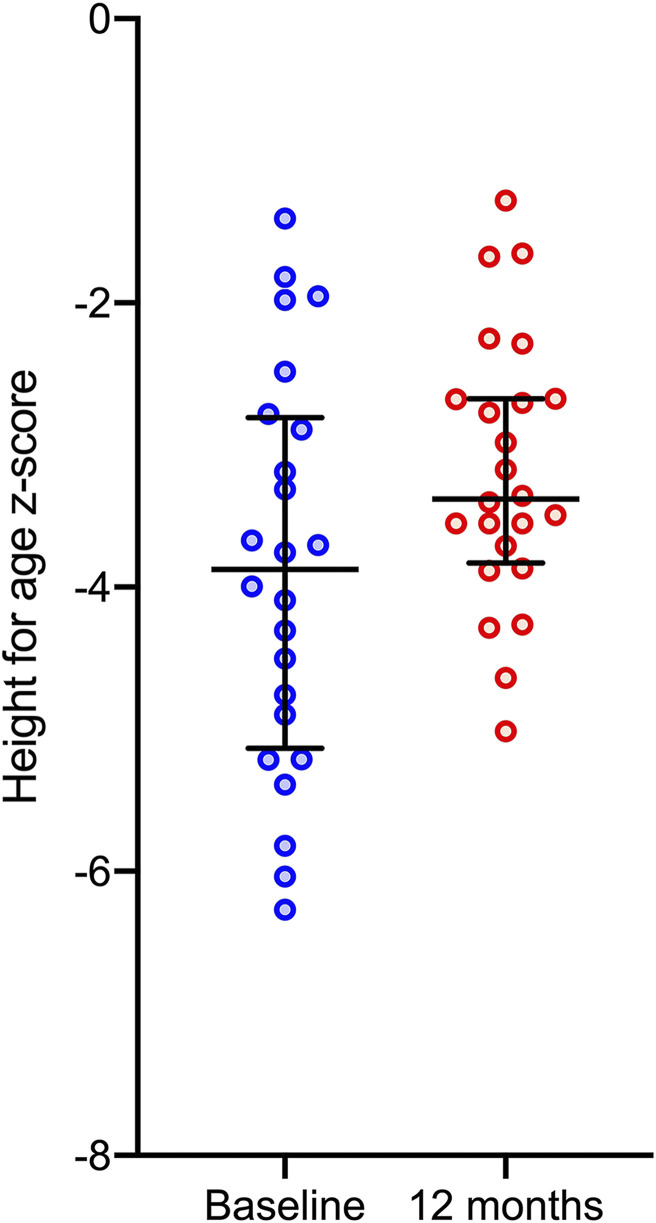
Height-for-age z-score for children at baseline and at 12-month follow-up, with the median (bar) and interquartile range (capped bars).

The children had a median age of 6 years (interquartile range [IQR], 5–8 years). Twelve of the 29 children had a first-degree relative included in the case series. All but one patient complained of joint pain and walking difficulty that started at a median age of 3 years (IQR, 2–4 years). Leg deformity (genu valgum, 72%; genu varum, 28%), antalgic gait (86%), and wrist enlargement (75%) were the most common abnormalities. Three patients (10%) were unable to walk. Other deformities included pectus carinatum (62%), rachitic rosary (17%), and teeth abnormalities (31%) (Table 1). Most patients were stunted (27 of 29, 93%) (Figures 2 and 3) and underweight (15 of 21, 71%). Ten of 25 children started walking after 18 months of age. Patients reported a diet composed primarily of rice, grains, and leafy vegetables, without food rich in vitamin D and/or calcium, such as fish, eggs, or milk products. Meat was consumed only rarely, after successful hunting. Fortified food was absent. Children did not avoid sun exposure unless their mobility was severely limited.

**Table 1:** Demographic and clinical findings (*N* = 29)

Characteristics	Data
Demographics	
Age at diagnosis, y; median (IQR) [range]	6 (5–8) [3–17]
Age at onset, y; median (IQR) [range]	3 (2–4) [2–15]
Male, *N* (%)	16 (55.2)
Female, *N* (%)	13 (44.8)
First-degree family relative with rickets, *N* (%)	12 (41.4)
Clinical findings, *N* (%)	
Joint tenderness of knee, ankle, or wrist	9 (24)
Bossing of the skull	5 (17)
Dental deformity	9 (31)
Wrist enlargement	22 (75)
Pectus excavatum or pectus carinatum	18 (62)
Rachitic rosary	5 (17)
Kyphosis	2 (6)
Leg deformity	29 (100)
Genu valgum	21 (72)
Genu varum	8 (28)
Antalgic gait	25 (86)
Unable to walk	3 (10)

IQR = interquartile range.

Serum biochemistry demonstrated a low 25-hydroxyvitamin D (25OHD) level of < 30 nmol/L in 22 of 29 (76%) children, a high parathyroid hormone PTH) level of > 65 pg/mL in 22 of 28 (79%) children, and an elevated alkaline phosphatase (ALP) level of > 800 UL in 28 of 29 children (97%). Fourteen children (48%) had low serum phosphate levels and six (21%) had low serum calcium levels (Table 2). Nonfasted venous blood samples were kept between 2 and 8°C, and were analyzed within 48 hours at a laboratory in Yangon. Serum concentrations of 25OHD and PTH were calculated using an electrochemiluminescence immunoassay (Roche cobas e411 analyzer, Roche Diagnostics International, Rotkreuz ZG, Switzerland).

**Table 2:** Biochemical findings at baseline and after treatment with vitamin D_3_ and calcium carbonate.

Biochemical tests	Pre-treatment (*N* = 29), median (IQR)	Post-treatment (*N* = 25), median (IQR)	Non-parametric Mann-Whitney test *P* value
Serum 25OHD; normal range, > 30 nmol/L	22.4 (13.8–29.9)	80.0 (53.7–98.7)	< 0.0001
Plasma PTH; normal range, 15–65 pg/mL	122.9 (71.5–223.6)	31.1 (18.6–52.7)	< 0.001
Serum ALP; normal range, < 800 UL	1,956 (1,204–2,892)	888 (794–1,034)	< 0.001
Serum phosphate; normal range, 1.29–2.26 nmol/L	1.34 (1.06–1.50)	1.22 (1.12–1.36)	0.396
Serum calcium; normal range, 2.00–2.50 nmol/L	2.15 (2.05–2.29)	2.30 (2.19–2.39)	0.004

25OHD = 25-hydroxyvitamin D; ALT = alkaline phosphatase; IQR = interquartile range; PTH = parathyroid hormone.

**Figure 4.**
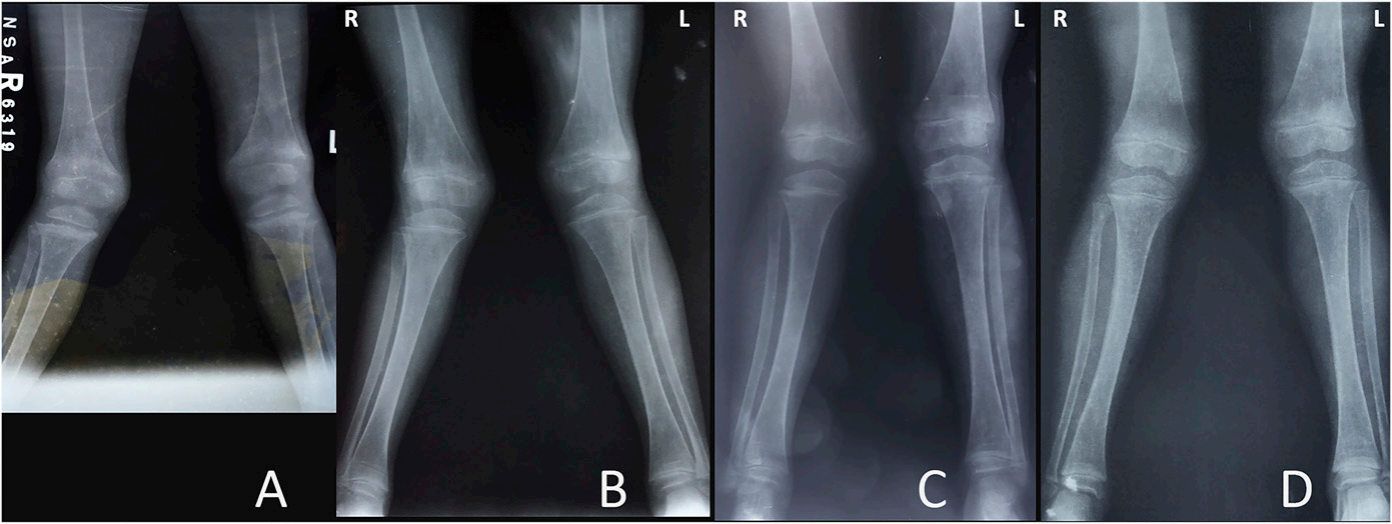
X-rays of a 6-year-old girl. (**A**) Pre-treatment X-ray shows severe widening/cupping of the metaphyseal growth plates with sclerosis, hypertrophic growth plates, bowing of the diaphyses, and diffuse osteopenia. (**B–D**) Post-treatment X-rays show a gradual straightening of the diaphysis, reduction in sclerosis at the epiphyses, and normalization of the distance between the distal metaphyses and epiphyses over time.

Because of the extreme remoteness of the children’s location, X-ray examination performed at the Township hospital was limited to five children. X-rays illustrated diffuse osteopenia, widening of the metaphyseal growth plates with sclerosis, hypertrophic growth plates, and bowing of the diaphyses (Figure 4).

**Figure 5.**
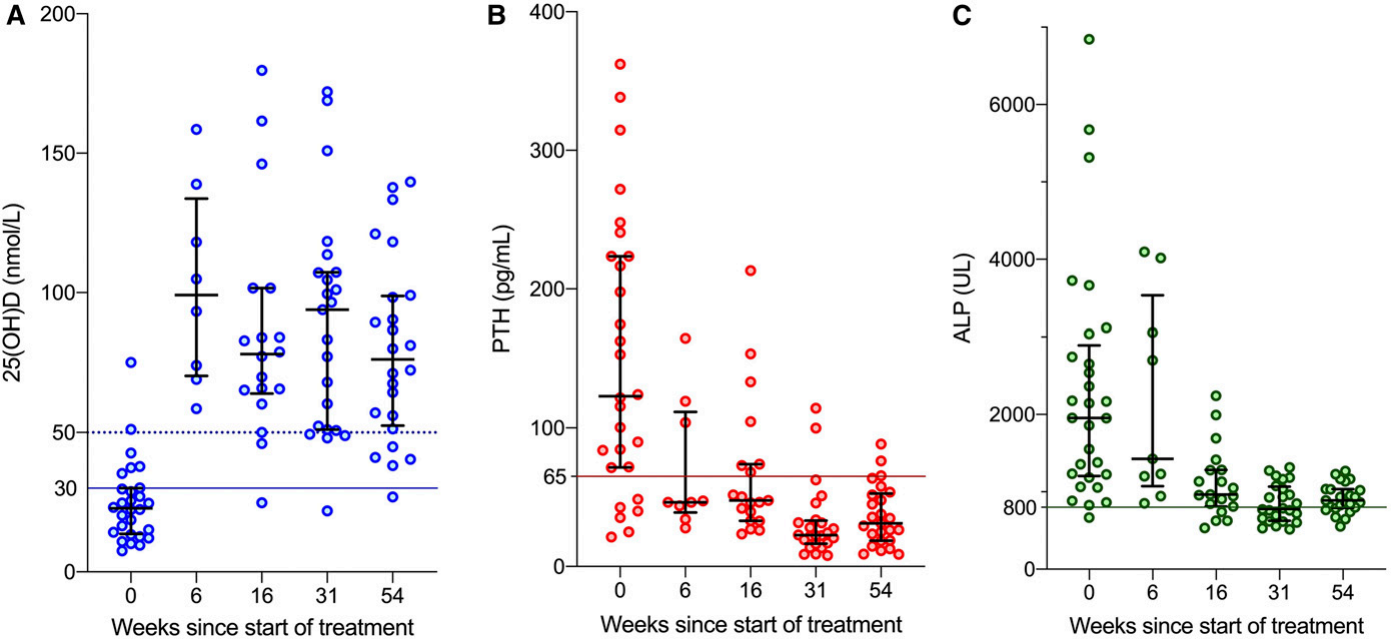
(**A**) Serum 25-hydroxyvitamin D (25(OH)D; blue) values at presentation and at follow-up after 1 year. (**B**) Parathyroid (PTH; red) values at presentation and at follow-up after 1 year. (**C**) Alkaline phosphatase (ALP; green) values at presentaTtion and at follow-up after 1 year. Individual patient values (small circles) are shown, along with median (bar) and interquartile range (capped bars). The dotted horizontal line in (**A**) on the *y* axis represents the lower limit of normal (50 nmol/L) and the continuous line on *y* axis represents the upper limit of depletion (30 nmol/L). The continuous lines in (**B**) and (**C**) represent the upper limit of normal range for PTH (65 pg/mL) and ALP (800 UL), respectively. Note that not all children had serum sampling at each follow-up.

A diagnosis of nutritional rickets with low 25OHD was made and the children were administered vitamin D_3_ (a loading dose of 100,000 IU, followed by 5,000 IU/d for 2 months and then 1,000 IU/d for 10 months) plus calcium carbonate (750 mg/d for children with a bodyweight of 10–19 kg and 1,500 mg/d for children ≥ 20 kg for 2 months, followed by 500 mg/d for all). The children were monitored every 2 to 3 months for 12 months. At 12 months, the children reported improved joint pain and mobility (93% and 96%, respectively). Two of 3 children who could not walk at all before, were able to walk. The third child had malunion and nonunion fractures at an early age and still could not walk. Considerable improvement was seen in biochemistry (Table 2 and Figure 5) and in radiography, in the five children who had undergone serial radiography (Figure 4). There was a significant improvement in height-for-age z-score: from –3.89 at baseline to –3.20 at 12 months (Wilcoxon’s signed rank test, *P* < 0.0001) (Figure 3).

## DISCUSSION

We describe a cluster of children with rickets who presented with severe, disabling symptoms and skeletal deformities; and low 25OHD, raised PTH, and raised ALP levels consistent with calcipenic rickets. Treatment with vitamin D_3_ and calcium carbonate resulted in considerable symptomatic, biochemical, and radiological improvement.

These children are the first reported cases of rickets in Myanmar for 120 years, following only a description of rickets in 19th-century Burma.^6^ In the neighboring countries of Bangladesh and India, rickets has been described repeatedly, with varying etiological factors depending on age and geographic location. Rickets in Bangladesh is more common after 2 years of age, and calcium deficiency is thought to be the primary etiological factor in the context of poor vitamin D status.^7–9^ In north India, dietary calcium deficiency was identified as the cause of rickets among young children (< 10 years), whereas rickets among adolescent girls was caused by vitamin D deficiency.^10^ In another study in north India, children (age, 6 months–5 years) with rickets experienced a better response after a combination of vitamin D plus calcium compared with vitamin D or calcium alone, indicating that a combination of vitamin D and calcium deficiency was causing rickets.^11^ In the tea plantations of east Assam, northeast India, close to the Naga region in Myanmar, of 16,274 screened children age 1 to 18 years, 44 (0.27%) had skeletal deformities consistent with nutritional rickets.^12^ Causative factors of rickets were not reported.

**Figure 6.**
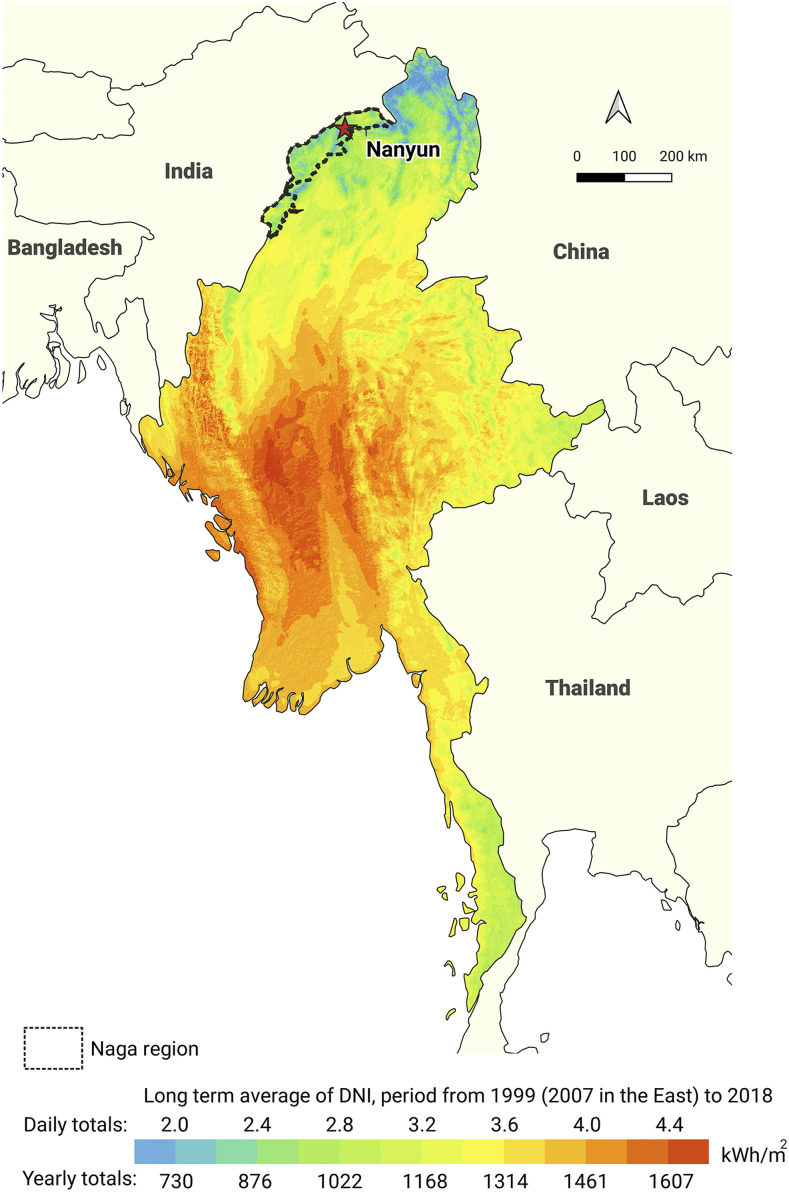
Direct normal irradiation in Myanmar. Long-term average of yearly totals.^14^

In Myanmar, it is generally assumed that ultraviolet B radiation is adequate to prevent vitamin D deficiency. However, the diet of the people in Naga is based predominantly on rice, grains, and leafy vegetables, and appears to be very low in vitamin D. Pregnant and lactating women in these communities could be vitamin D deficient. Breast milk contains very little vitamin D, and in the absence of vitamin D supplementation, prolonged breastfeeding (median duration, 20 months in this case series) likely contributed to low vitamin D levels in infants.^13^ The mountainous Naga region is often shrouded in clouds and sunlight is among the lowest in the country (Figure 6).^14^ Naga children do not habitually avoid sun unless their mobility is restricted. As several children reported a late start in walking, limited ultraviolet exposure may have played a contributing factor to vitamin D deficiency. After breastfeeding, the typical diet was low in vitamin D and calcium, and high in phytates. Calcium deficiency could be a causative factor, in combination with vitamin D deficiency or on its own. This could explain the late onset of recognized symptoms, at 3 years of age, after the start of weaning. The age of onset was similar to a study conducted in southeast Bangladesh, where rickets was predominantly the result of a dietary calcium deficiency in the context of a chronically low vitamin D status.^10^ However, the serum calcium levels of the Bangladeshi children with active rickets were less than in our cohort, possibly suggesting an alternative etiology.^10^

We acknowledge the limitations inherent to case series study design. Additional limitations of this study include the limited access to radiography, remote laboratory analysis, and lack of a comprehensive dietary assessment. Additional causative factors, such as environmental heavy metals or fluoride exposure, that may have contributed to the clinical syndrome require additional investigation.^15^ Furthermore, a genetic abnormality of vitamin D biosynthesis, such as a mutation of 25OHD (CYP2R1), although extremely rare, needs to be excluded, particularly given the apparent clustering within families.^16^

During the 12-month follow-up of the 29 children described in our study, an additional 307 children were clinically diagnosed and treated for rickets in Naga, making rickets an important public health problem in this region. Fortifying staple foods with vitamin D, and promoting the intake of indigenous food sources rich in calcium or vitamin D and calcium supplements may be required to prevent this potentially devastating disease.

## CONCLUSION

Calcipenic rickets is an important problem for children in the Naga region. Several factors made these children at risk. First, the local diet appears to be low in vitamin D, potentially leading to maternal deficiency and low vitamin D levels of newborns and breastfed children. Second, the diet also appears to be low in calcium and high in phytates, potentially causing calcium deficiency. Third, breastfeeding is generally prolonged. Fourth, sunlight is relatively low in this region. Treatment with vitamin D and calcium improved symptoms and reversed bone deformities. Further research is urgently required to understand more completely the etiology of this neglected and devastating disease. This understanding can determine the specific interventions required.

## References

[1] World Health Organization, 2019. *Nutritional Rickets: A Review of Disease Burden, Causes, Diagnosis, Prevention and Treatment*. Geneva, Switzerland: WHO. Available at: https://www.who.int/publications/i/item/9789241516587. Accessed November 2, 2020.

[2] Agarwal A Gulati D Rath S Walia M, 2009. Rickets: a cause of delayed walking in toddlers. Indian J Pediatr 76: 269–272. 19347666 10.1007/s12098-009-0052-y

[3] Pettifor JM, 2004. Nutritional rickets: deficiency of vitamin D, calcium, or both? Am J Clin Nutr 80: 1725–1729. 10.1093/ajcn/80.6.1725S 15585795

[4] Mendes MM Hart KH Botelho PB Lanham-New SA, 2018. Vitamin D status in the tropics: is sunlight exposure the main determinant? Nutr Bull 43: 428–434.

[5] Edidin DV Levitsky LL Schey W Dumbovic N Campos A, 1980. Resurgence of nutritional rickets associated with breast-feeding and special dietary practices. Pediatrics 65: 232–235. 7354968

[6] Henderson AH, 1899. Rickets in India and Burma. Ind Med Gaz 34: 268. PMC5145414 29002260

[7] Craviari T Pettifor JM Thacher TD Meisner C Arnaud J Fischer PR, Rickets Convergence Group, 2008. Rickets: an overview and future directions, with special reference to Bangladesh: a summary of the Rickets Convergence Group Meeting, Dhaka, 26–27 January 2006. J Health Popul Nutr 26: 112–121. 18637536 PMC2740674

[8] Ahmed S Goldberg GR Raqib R Roy SK Haque S Braithwaite VS Pettifor JM Prentice A, 2020. Aetiology of nutritional rickets in rural Bangladeshi children. Bone 136: 115357. 32276153 10.1016/j.bone.2020.115357 PMC7262584

[9] Combs GF Hassan N Dellagana N Staab D Fischer P Hunt C Watts J, 2008. Apparent efficacy of food-based calcium supplementation in preventing rickets in Bangladesh. Biol Trace Elem Res 121: 193–204. 18180882 10.1007/s12011-007-8053-z

[10] Balasubramanian K Rajeswari J Gulab A Govil YC Agarwal AK Kumar A Bhatia V, 2003. Varying role of vitamin D deficiency in the etiology of rickets in young children vs. adolescents in northern India. J Trop Pediatr 49: 201–206. 12929879 10.1093/tropej/49.4.201

[11] Aggarwal V Seth A Marwaha RK Sharma B Sonkar P Singh S Aneja S, 2013. Management of nutritional rickets in Indian children: a randomized controlled trial. J Trop Pediatr 59: 127–133. 23104564 10.1093/tropej/fms058

[12] Chabra T Tahbildar P Sharma A Boruah S Mahajan R Raje A, 2016. Prevalence of skeletal deformity due to nutritional rickets in children between 1 and 18 years in tea garden community. J Clin Orthop Trauma 7: 86–89. 27182144 10.1016/j.jcot.2016.01.005 PMC4857165

[13] Streym SV Højskov CS Møller UK Heickendorff L Vestergaard P Mosekilde L Rejnmark L, 2016. Vitamin D content in human breast milk: a 9-mo follow-up study. Am J Clin Nutr 103: 107–114. 26675779 10.3945/ajcn.115.115105

[14] The World Bank, 2019. *Global Solar Atlas 2.0, Solar Resource Data: Solargis*. Available at: https://solargis.com/maps-and-gis-data/download/myanmar. Accessed April 25, 2021.

[15] Qin X Wang S Yu M Zhang L Li X Zuo Z Zhang X Wang L, 2009. Child skeletal fluorosis from indoor burning of coal in southwestern China. J Environ Public Health 2009: 969764. 20041010 10.1155/2009/969764 PMC2778178

[16] Thacher TD Fischer PR Singh RJ Roizen J Levine MA, 2015. CYP2R1 mutations impair generation of 25-hydroxyvitamin D and cause an atypical form of vitamin D deficiency. J Clin Endocrinol Metab 100: E1005–E1013. 25942481 10.1210/jc.2015-1746 PMC4490307

